# Multidisciplinary approach to scheduling surgery for diabetic foot: a case report

**DOI:** 10.1186/s12891-019-2522-3

**Published:** 2019-04-12

**Authors:** Guangyu Wang, Leiyong Wang, Yu Wang, Xuying Xu

**Affiliations:** Department of Chinese Medicine Surgery, Beijing Hospital of Traditional Chinese Medicine, Affiliated to the Capital Medical University, No.23, Back Road of the Art Gallery, Dongcheng District, Beijing, 100010 China

**Keywords:** Multidisciplinary collaboration, Diabetic foot ulcer, Timing, Interventional therapy

## Abstract

**Background:**

The treatment of diabetic foot ulcers in this case is complex and multidisciplinary, and an interdisciplinary team is extremely beneficial.

**Case presentation:**

We performed the intervention on an old type 2 diabetes patient with poor health, whose left toes were severely necrotic. Surgery, including debridement and patella truncation, had positive effects on lower extremity circulation, infection control, cavity treatment, bone destruction, surgical debridement, recovery of foot function, and nursing. After 5 months, the patient’s foot ulcer had healed, and walking function was preserved.

**Conclusions:**

Scheduling interventional surgery and debridement are the key point in a complicated diabetic foot ulcers case, and multidisciplinary collaboration in treatment of diabetic foot is significantly important.

## Introduction

In China, in 2010, the prevalence of diabetes was 11.6% [1], while the proportion of diabetic patients > 50 years old with lower extremity arterial disease was 19.5% [2]; the proportion of diabetic patients > 60 years old with lower extremity arterial disease was 35.4% [3]. The incidence of new foot ulcers in Chinese patients with diabetes during the period from 2010 to 2011 was 8.1%, and the incidence of new ulcers in patients with diabetic foot ulcers within that period was 31.6% [4]. Diabetic foot ulcers have gradually become a challenge in clinical therapy and a leading cause of hospitalization among patients with diabetes [5].

The medical community has reached a consensus in favor of multidisciplinary collaboration in treatment of diabetic foot [6–9]. The complexity of treatment for diabetic foot determines the importance of optimizing the timing of multi-disciplinary and sequential treatments. This case presentation describes a complicated process of treatment for an elderly patient with diabetic foot ulcers, who had signs of poor baseline physiology. The wounds healed, and foot function was maximally preserved.

## Case report

A 68-year-old man with 21-year history of type 2 diabetes presented with an ulcer on the left heel. Height was 162 cm; body weight was 69 kg; body mass index was 26.3. The patient had been diagnosed with lower extremity atherosclerotic obliterans 7 years earlier. The left lower limb has been numb for 6 years, with intermittent claudication and rest pain for 1 year. The patient reported that his sleep was affected, but his degree of pain was decreased with the intermittent use of analgesic agents. For the left lower limb with claudication, walking distance was 90 m.

The patient was hospitalized on 10 July 2017. Twenty days before hospitalization, irritation and pain developed on the lateral skin of the toes of the left foot, with no obvious inducement. Purulent exudate was observed after skin ulceration, and the patient’s body temperature increased to 39.5 °C. During hospitalization, the five toes of the left foot were black, necrotic, and associated with aggravated rest pain. Although the dose of oral analgesics was increased, the patient’s pain was not relieved, and his sleep was severely affected.

**Fig. 1:**
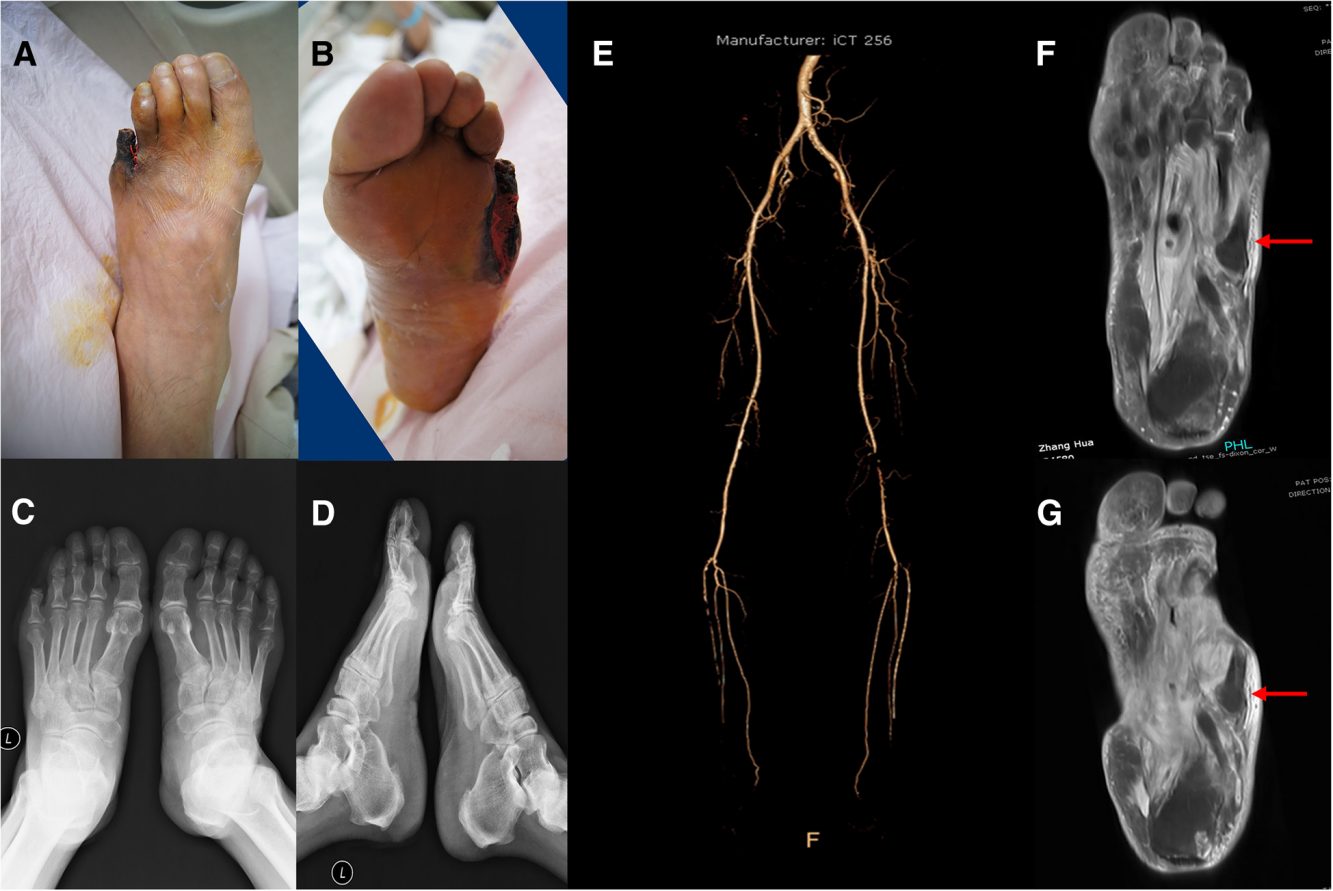
Diagnostic assessment of the diabetic foot ulcer. **ab**, 10/7/2017; **cd**, foot X-ray; **e**, Lower extremity CTA. 13/7/2017. **f**, foot MRA,14/7/2017. **g**, foot MRA, 4/9/2017

The patient’s appearance on initial evaluation is shown in Fig. 1a and b. The five toes of the left foot were almost entirely black and necrotic. The skin extending from the bottom of the foot to the 5th metatarsophalangeal joint was red and swollen, with obvious tenderness; skin temperature was normal. The muscles of the left foot had clearly atrophied; the skin was thin, bright, and hypertonic. Incision and drainage (approx. 5.0-cm long) was immediately performed between the 4th and 5th toes. Necrotic tissue, minimal purulent exudation, and limited bleeding were observed.

The diagnostic results on obtained on July 13 are shown.Secretion cultures displayed *Pseudomonas aeruginosa* and *Staphylococcus aureus*. X-ray film showed in Fig. 1c and d revealed no obvious destruction in foot bone. The lower limb computed tomographic angiography (CTA) is shown in Fig. 1e.

**Fig. 2:**
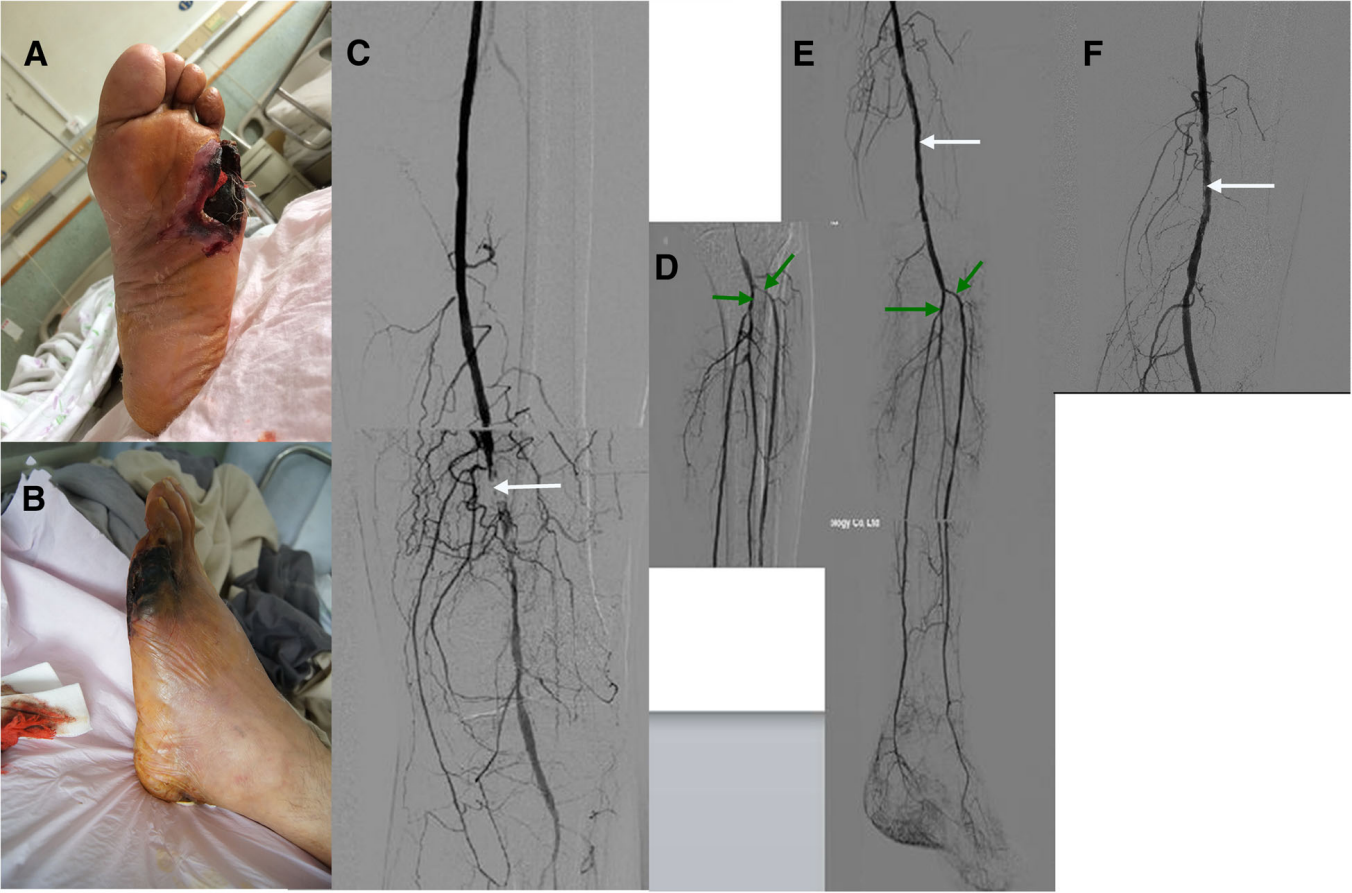
Administration of first therapeutic intervention. **ab**, 20/7/2017; **cde**, 18/7/2017; **f**, 20/7/2017. **c** white arrow is the superficial femoral artery thrombus. **e** white arrow is the balloon dilatation; **f** white arrow is the contrast artery after catheter thrombolysis; **d** green arrow is the narrow anterior iliac artery and the iliac artery; **e** green arrow is the ball The anterior iliac artery and the iliac artery after expansion of the capsule

**Fig. 3:**
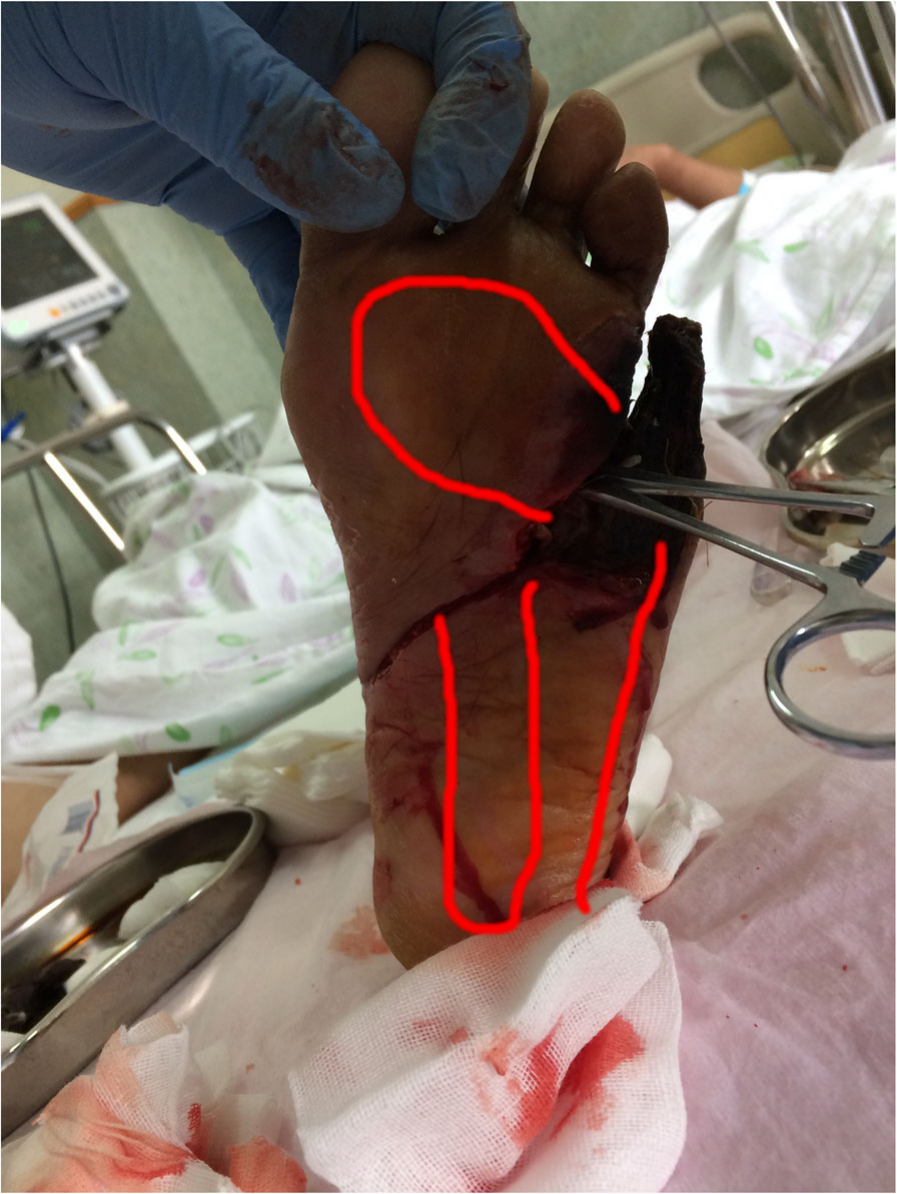
Subcavities found in the left foot. 20/7/2017

The magnetic resonance imaging (MRI) of the foot obtained on July 14th is shown in Fig. 1f and g. Subcutaneous inflammatory tissue in the lateral 5th humerus bone was confirmed as an infectious submerged cavity (Fig. 3). The first incision performed at the bedside resulted in limited purulent exudation, as well as decreased local tension and reduced foot swelling. However, progressive necrosis of the skin margin was noted (Fig. 2a and b).

On July 17, basal therapy consisted in controlling blood sugar with insulin (58 units/day). The initial antimicrobial application of ceftazidime infusion (2 g, twice daily) was replaced by the sensitive drug Sulperazon (Cefoperazone Sodium and Sulbactam Sodium for Injection, Pfizer), according to the bacterial culture result of the secretion, and the application of antibiotics was stopped after July 22. The patient showed improved circulation and blood pressure. Treatment was maintained with protein and iron supplements.

On July 18th, interventional therapy was performed on the lower limb. The balloon was expanded in the superficial femoral artery, anterior tibia artery, iliac artery, and tendon. A thrombus had formed in the lower segment of the superficial femoral artery. The catheter for thrombolysis was left in place until July 20 (Fig. 2c, d, e and f). Skin temperature in the left foot increased, and swelling extended to the center of the foot. The amount of purulent exudation increased, as did pain and body temperature. The foot was therefore incised and drained, once more. Multiple subcavities were found (Fig. 3).

**Fig. 4:**
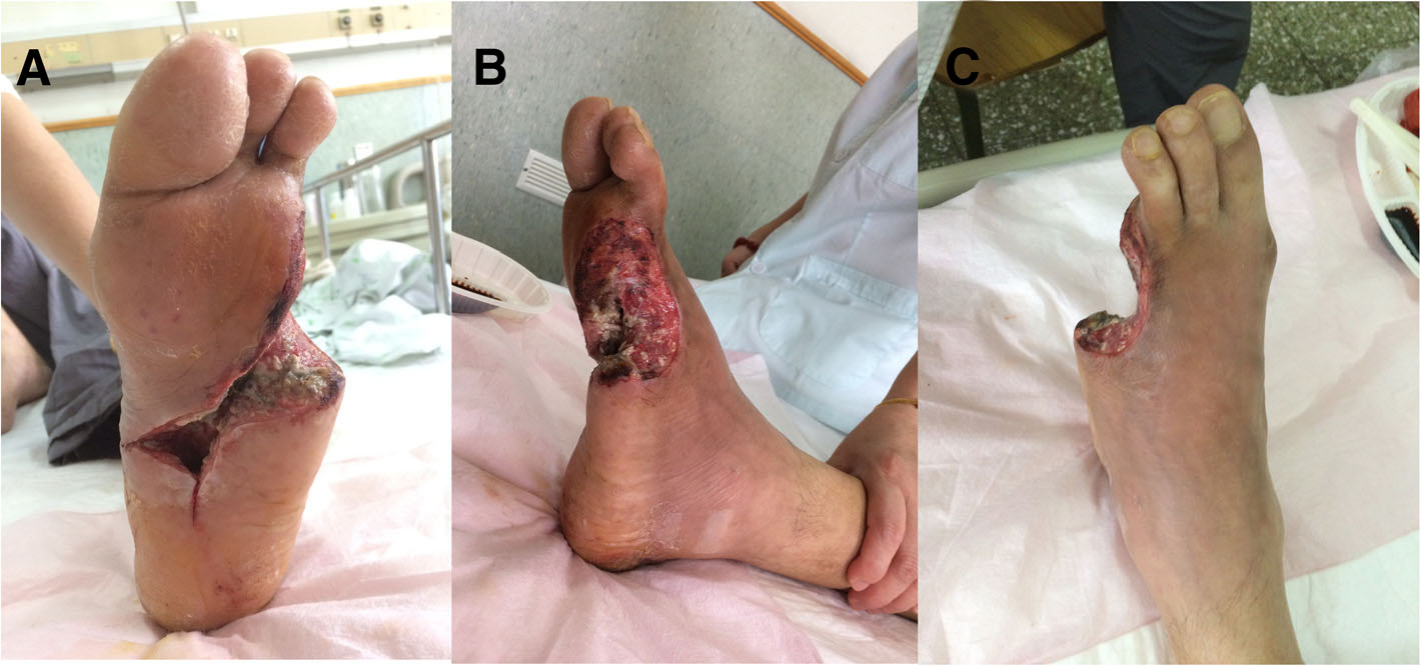
Results of patella truncation and debridement. **abc**: 24/7/2017

On July 21st, we performed patella truncation and debridement on toes 4 and 5. The procedure was performed in the operating room, with the patient under sciatic nerve block. Results are presented in Fig. 4. Submerged cavities were filled with povidone-iodine gauze. The amount of nonviable tissue in the submerged cavity gradually decreased. The submerged space became shallow, and the granulation filled. All subsequent treatments were performed in the outpatient department.

**Fig. 5:**
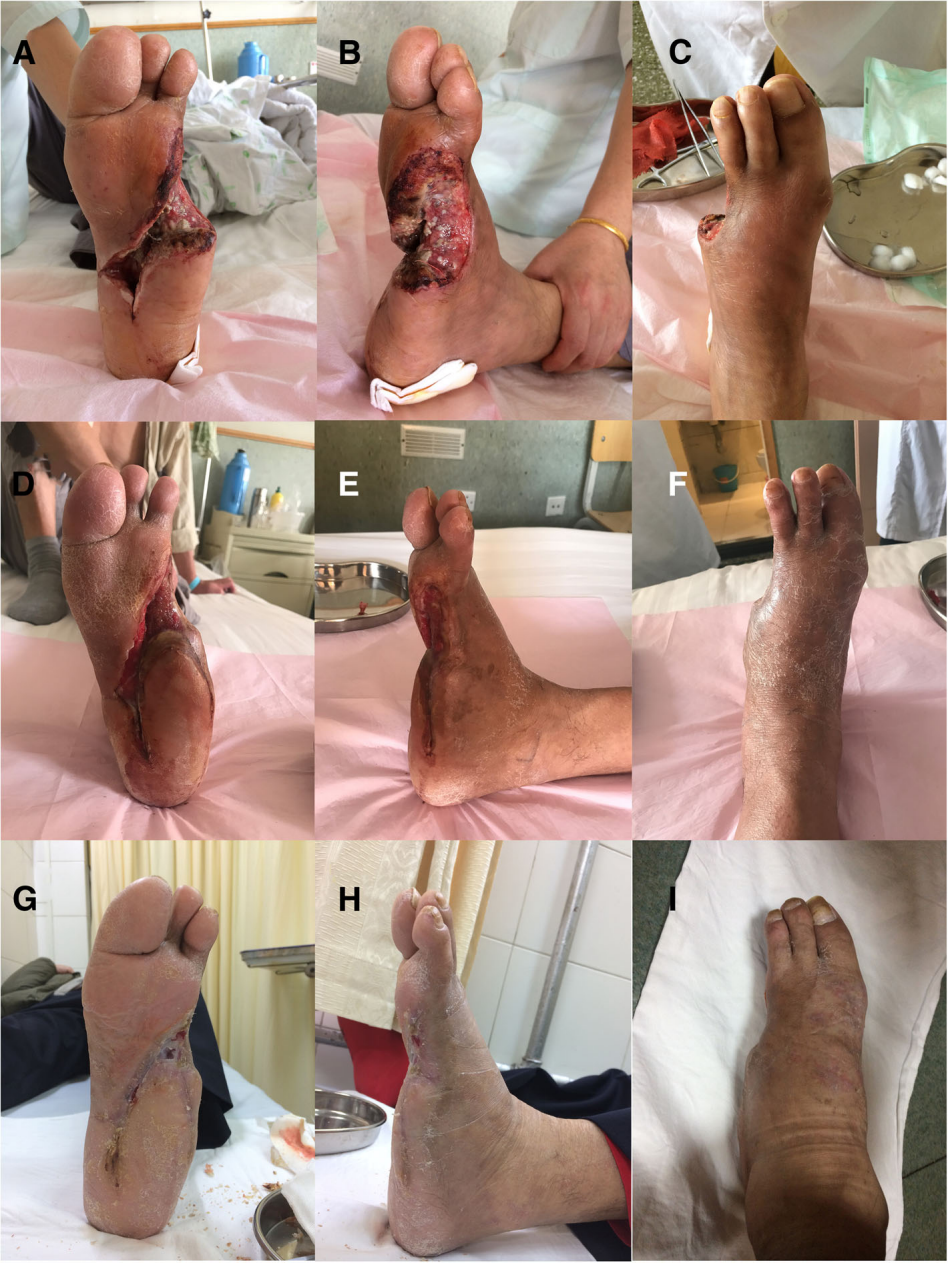
Follow-up of the surgical intervention and outcomes. **abc**: 25/7/2017. **def**: 30/9/2017. **ghi**: 19/11/2017

From July 25th to September 30th, the left foot plaster was fixed at a functional position and applied intermittently (Fig. 5a, b, c, d, e and f). After October 1st, the plaster was removed. The patient was given diabetic shoes, and walking was guided. The first signs of wound healing were observed on November 19 (Fig. 5g, h and i).

## Discussion

### Debridement schedule

In order to minimize adverse effects, local debridement must be initiated in an expedient fashion. In the case presented above, the foot was severely ischemic and therefore could not support local blood transport after incision. In addition, ​​infection easily spreads along the physiological cavity extending from the little toe. Efforts to avoid skin necrosis and wound expansion typically render comprehensive and meticulous debridement impossible.

After the initial treatment, we continued with nibbling debridement, in order to preserve as much residual tissue as possible. Debridement can be performed more thoroughly after blood transport is improved. The surgeon must continue to avoid over-injury, in order to restrict the area of infection. After the second episode of debridement [9], the opening of the cavity was patent and incompletely dissected (Fig. 4). It is also possible to cut into the subcavities of the heel (Fig. 5d and g) and the lateral portion of the 5 tibia (Fig. 5e and h).

### Intervention

Endovascular treatment may improve the blood supply to the lower limbs and create conditions conducive to thorough debridement and healing [10]. Such treatment should be initiated once the local infection is restricted, and the patient is stable. However, after the intervention, improved wound circulation increases the inflammatory response. In this case, the patient underwent substantial debridement during thrombolysis, when it was discovered that the infection had spread along physiological lacunae (Figs. 3 and 4). Blood supply to the lower extremities was not considered sufficient for skin grafting.

### Debridement

The term “nibbling debridement” denotes debridement that is gradual in spatial and temporal dimensions. Debridement is generally used to treat diabetic feet, which often have insufficient blood supply. The surgeons attempted to preserve as much tissue as possible, with the extent of debridement determined by guidelines for treatment during the infection, proliferative, and healing phases. The sites of debridement included: necrotic skin margins, fascia, deeper cavities, residual tendon and inactivated tissue in shallow lances, and hyperplastic granulation (during the later stages of treatment).

### Preservation of foot function

In the early stage of treatment, it is necessary to fix the functional position in order to avoid supporting and walking. Such precautions are necessary to avoid the flexion and extension of large joints. During the middle stages of treatment, joint activity may increase slightly. Once the plaster is fixed, the foot may be supported on the floor. However, walking should be forbidden, in order to avoid movement between tissues. During the latter stages of treatment, when healing is clearly underway, the patient should be given shoes tailored specifically for diabetic feet, to provide support during walking [11]. Training sessions to improve function of the lower extremities should be completed with increasing frequency, but the use of conventional shoes is prohibited.

## Conclusions

The treatment of diabetic foot ulcers is extremely complicated. During the initial stages of diagnosis, a multidisciplinary team should make treatment recommendations based on the severity of ischemia and the degree of infection. If the foot cannot be preserved, thorough debridement may be performed during the initial stages of treatment to reduce adverse effects on the body. In combination with standard treatment, conditions for amputation should be achieved in as expedient a fashion as possible. Multi-disciplinary cooperation and optimization of the treatment schedule are necessary if there is even a remote possibility of preserving any portion of the foot or toes.

## Abbreviation

CTA: Computed tomographic angiography
